# Increased Polyamine Intake Inhibits Age-Associated Alteration in Global DNA Methylation and 1,2-Dimethylhydrazine-Induced Tumorigenesis

**DOI:** 10.1371/journal.pone.0064357

**Published:** 2013-05-16

**Authors:** Kuniyasu Soda, Yoshihiko Kano, Fumihiro Chiba, Kei Koizumi, Yuichiro Miyaki

**Affiliations:** 1 Department of Cardiovascular Research Institute, Saitama Medical Center, Jichi Medical University, Saitama-city, Saitama, Japan; 2 Departments of Surgery, Saitama Medical Center, Jichi Medical University, Saitama-city, Saitama, Japan; 3 First Department of Surgery, Hamamatsu University School of Medicine, Hamamatsu, Shizuoka, Japan; University of Washington, United States of America

## Abstract

Polyamines (spermine and spermidine) play many important roles in cellular function and are supplied from the intestinal lumen. We have shown that continuous high polyamine intake inhibits age-associated pathologies in mice. The mechanism by which polyamines elicit these effects was examined. Twenty-four week old Jc1:ICR male mice were fed one of three experimental chows containing different polyamine concentrations. Lifetime intake of high polyamine chow, which had a polyamine content approximately three times higher than regular chow, elevated polyamine concentrations in whole blood, suppressed age-associated increases in pro-inflammatory status, decreased age-associated pathological changes, inhibited age-associated global alteration in DNA methylation status and reduced the mortality in aged mice. Exogenous spermine augmented DNA methyltransferase activity in Jurkat and HT-29 cells and inhibited polyamine deficiency-induced global alteration in DNA methylation status *in vitro*. In addition, increased polyamine intake was associated with a decreased incidence of colon tumors in BALB/c mice after 1,2-demethylhydrazine administration; 12 mice (60%) in the low polyamine group developed tumors, compared with only 5 mice (25%) in the high polyamine group (Fisher's exact probability = 0.027, *p* = 0.025). However, increased polyamine intake accelerated the growth of established tumors; maximal tumor diameter in the Low and High groups was 3.85±0.90 mm and 5.50±1.93 mm, respectively (Mann-Whitney test, *p* = 0.039). Spermine seems to play important roles in inhibiting age-associated and polyamine-deficient induced abnormal gene methylation as well as pathological changes including tumorigenesis.

## Introduction

**Figure 1:**
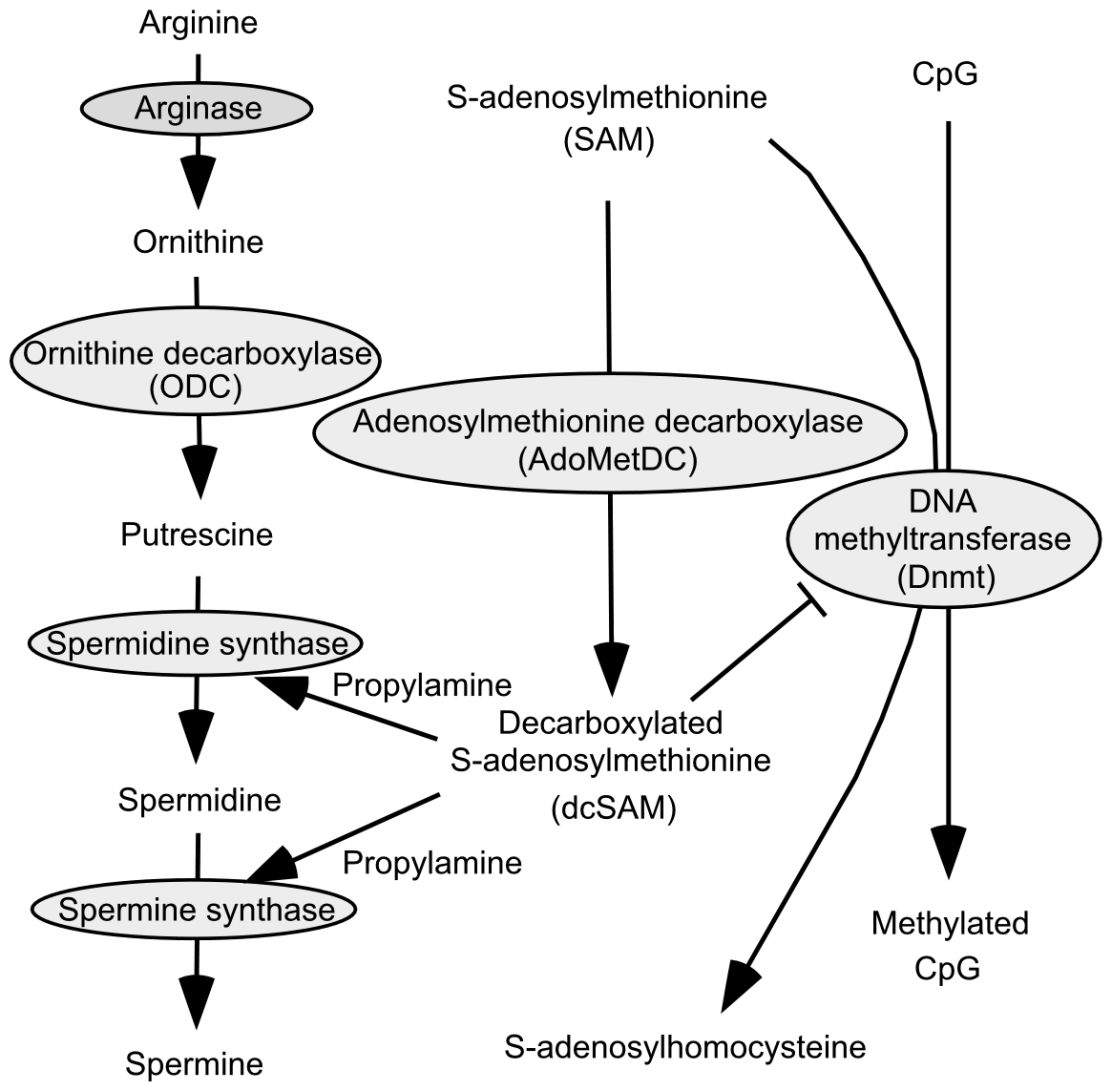
Metabolic pathway for polyamine synthesis, Dnmt activity and gene methylation. SAM is a methyl donor, and dcSAM inhibits Dnmt activity. ODC = Ornithine decarboxylase; AdoMetDC = Adenosylmethionine decarboxylase; SAM = S-adenosylmethionine; dcSAM = Decarboxylate S-adenosylmethionine; Dnmt = DNA methyltransferase.

The polyamines spermine and spermidine (Figure 1) are essential for cell growth and differentiation and have many biological activities that may contribute to inhibition of age-associated pathological changes, such as anti-inflammatory and anti-oxidant properties, free radical scavenger activity and general protection of the genome and cells from various harmful stimuli [1], [2], [3], [4], [5], [6]. Although enzymatic activity essential for polyamine synthesis decreases with aging, cells can take up exogenous polyamines, and the importance of dietary polyamines has increasingly been recognized [7]. For example, long-term, but not short term, increased consumption of polyamine-rich food has been shown to gradually increase blood polyamine concentration in humans and mice [8], [9], [10].

The present study was designed to follow up on our previous study showing that polyamines, and especially spermine, specifically suppress the age-associated increase in expression and activity of lymphocyte function associated antigen 1 (LFA-1 = CD11a/CD18) in peripheral blood mononuclear cells [2]. LFA-1 is one of the membrane–bound proteins that play a crucial role not only in cell adhesion but also in mediating cell-cell interactions, and their enhanced expression results in an augmented capability of cell adhesion and activation [11], [12]. The adhesion and activation of immune cells mediates inflammation [13], [14], therefore, the sustained overexpression of LFA-1 of the immune cells, such as PBMCs, in the elderly [15], [16] is considered to contribute the progression of chronic inflammatory diseases [2]. Chronic silent inflammation is implicated in many age-associated diseases [17], and therefore, this finding of suppression of age-associated increased pro-inflammatory status (as indicated by increased LFA-1 expression) by polyamines prompted us to further investigate the effects of polyamines on age-associated pathological alterations.

Recent studies have shown that spermidine extends the lifespan of yeast, flies, worms and human cells [18]. However, the importance of polyamines appears to differ among organisms; for example, mice lacking spermine synthase have a variety of defects including significant growth retardation, a very short life span, deafness, and neurological deficits [5], [19]. Spermine is needed for normal growth in mammals, but not in yeast or plants, suggesting that spermine is more important for mammals than for many other organisms [20]. Another interesting aspect of the effects of dietary polyamines is that long-term administration of a high polyamine diet (mixture of spermine, spermidine, and putrescine) elevated blood spermine levels, but not spermidine levels [8]. Moreover, the effects of spermine on LFA-1 suppression were shown to be more potent than those of spermidine [2]. Therefore, in the present study, we employed spermine to investigate the biological activities *in vitro*.

In the present study, we further examined whether the increased polyamine intake actually suppress LFA-1 expression in aged mice. In addition, in order to investigate the biological background that may lead suppression of age-associated pathologies, we investigated the effect of spermine on methylation status of whole genome of murine organs and cultured cell, because polyamine metabolism has a close association with gene methylation. Moreover, the result that spermine suppressed polyamine-deficiency induced global alteration in DNA methylation status prompted us to test the possible role of polyamine on inhibiting 1,2-dimethylhydrazine (DMH) induced neoplastic growth in the large intestine of mice.

## Materials and Methods

### Experimental chows

The experimental design was approved by the Institutional Review Board of Jichi Medical University, and all animal procedures followed the Principles of Laboratory Animal Care. Experimental chows were prepared by adding synthetic spermine (Wako Pure Chemical Industries Ltd., Osaka, Japan), spermidine (Sigma Chemical. Co., St. Louis, MO) and putrescine, a diamine, to the base “ = low” experimental chow prepared by Nippon Clea Co. (Tokyo, Japan). The concentrations (w/w) of spermine, spermidine and putrescine were 0.002 %, 0.008 % and 0.002 %, respectively, for the moderate polyamine chow (defined as “normal polyamine chow” in the previous report [9].), and 0.015 %, 0.06 % and 0.015 %, respectively, for the high polyamine chow. Details of the composition of the three experimental chows are shown in a previous report [9]. The mixture was pelleted, and concentrations of spermine, spermidine and putrescine as measured by high-performance liquid chromatography (HPLC) were 143, 224 and 496 nmol/g for the low polyamine chow, 160, 434 and 625 nmol/g for the moderate polyamine chow, and 374, 1540 and 1075 nmol/g for the high polyamine chow, respectively [9].

### Animals

Eight-week-old male Jc1:ICR mice were purchased from Nippon Clea Co. After 2 weeks of acclimatization, 4-5 mice per cage were randomly divided into 3 groups (the High, Moderate and Low polyamine chow groups), and housed in temperature-controlled cages supplied with high efficiency particulate-arresting filtered air. Mice had *ad libitum* access to standard rodent laboratory chow until 24 weeks of age, and to the experimental chow thereafter. Body weight was measured every other week at 10 am, and survival was checked every other day. When mice bred for blood sampling reached 80 weeks of age, blood sampling was performed to determine blood polyamine levels and LFA-1 expression levels. At the time of blood sampling, any diseased mice, such as those that appeared debilitated or with obvious tumors on the body surface or in the organs and tissues that may affect blood polyamine levels were excluded. Blood was drawn from the right atrium under pentobarbital anesthesia (60 µg/g body weight). When mice bred for histological and genetic examinations reached 88 weeks of age, the survival study was concluded, and animals were euthanized to extract organs and tissues. The kidney, liver, colon, heart and gastrocnemius muscle were extirpated and examined histologically, and kidneys were used for DNA methylation microarray analysis.

For experiments testing the effects of increased polyamine intake on neoplastic growth, 4-week old male mice (BALB/c) were housed in cages and had *ad libitum* access to standard laboratory chow. After one week of acclimatization, the 80 mice were divided into 2 groups: the high polyamine and low polyamine chow groups. Half the mice in each group (20 mice) were administered 20 mg/kg body weight (BW) of DMH (2.0 mg/ml in PBS) subcutaneously for 12 consecutive weeks while the remaining mice were injected with the same volume of PBS. After 25 weeks of experimental chow feeding (42 weeks of age), mice were euthanized and examined for neoplasms, especially in the large intestine.

### Cell culture studies

To exclude the effects of cytotoxic substances (aldehydes and hydrogen peroxide) produced by serum amine oxidase activity present in fetal bovine serum but not in the human body, the culture medium was supplemented with human serum. Jurkat and HT-29 cells were used to test the effects of polyamines on DNA methylation and DNA methyltransferase activity. Cells were cultured in RPMI-1640 supplemented with 10 % heat-inactivated human serum (Cosmo Bio Co., LTD., Tokyo, Japan) and 0.01 % Penicillin-Streptomycin (Invitrogen Corp., CA, USA) at a concentration of 3.0×10^5^ cells/ml for 72 hours. To deplete polyamines, alpha-d, l- difluoromethylornithine hydrochloride (DFMO) (ALEXIS Biochemicals Co., Lausen, Switzerland) was added to the culture medium at a final concentration of 3 mM. Our previous reports have shown that the biological activity of spermine is more potent than that of spermidine, and that serum spermine concentrations are significantly increased by increased polyamine intake in humans. Therefore, spermine was used for our experiments and was added to the culture medium containing DFMO at a final concentration of 500 µM. The final concentrations of DFMO and spermine were shown in preliminary experiments not to elicit any cytotoxic effects on cultured cells.

### Determination of polyamine concentrations

Whole blood samples were stored at −80°C in EDTA-coated tubes. To measure polyamine content, each sample was twice thawed and sonicated for 5 min and centrifuged at 18,000×*g* for 10 min. Each supernatant (100 µl) was then transferred to a new microfuge tube containing 20% trichloroacetic acid (100 µl) with 20 µM N-(3-aminopropyl) cadaverine used as an internal standard. After centrifugation at 18,000×*g* for 10 min, each supernatant was collected and stored at −20°C until measurement by HPLC (HPLC, LC-20AB, Shimadzu Corporation, Kyoto). Jurkat cells and HT-29 cells cultured for 72 hours were harvested and washed three times in excess PBS(-) to remove extracellular polyamines. One million cells were resuspended in 0.6 M perchloric acid, and then degraded by sonication (Eyela Ultrasonic Cleaner, 750 w, 30 sec, Tokyo Rikaikai Co. Ltd., Tokyo) and vigorous vortexing. Three hundred microliters of dansyl chloride containing 10 mg/ml acetone, 40 µM 1,7-diamninoheptane and saturated sodium carbonate solution were added to the lysate. After a 15 min of heat incubation at 70°C, 25 µl of proline solution in water (100 mg/ml) and 500 µl of toluene were added. The dried supernatant phase of the lysate was dissolved in 500 µM acetonitrile.

Concentrations of spermine and spermidine were measured by HPLC using Capcell Pak C18 MG (Shiseido Co. Ltd., Tokyo). HPLC was carried out as described in previous reports [8], [9].

### Flow cytometry

A FACSCalibur flow cytometer (BD Biosciences) was used to quantify CD11a expression in lymphocyte and monocyte populations. Thirty to fifty thousand cells gated in the lymphocyte and monocyte light scattered regions were analyzed. A FACScan flow cytometer (FACSCalibur, Japan Becton Dickinson, Tokyo, Japan) with analysis software (CeELLQuest) was used for analysis. Preparation of cells was carried out as described in a previous report [2].

### DNA methyltransferase (Dnmt) activity assay

The EpiQuik Nuclear Extraction Kit I and EpiQuik DNA Methyltransferase Activity/Inhibition Assay Kits (Epigentek Group Inc.) were used according to the manufacturer's protocols to measure Dnmt activity under various experimental conditions in Jurkat and HT-29 cells. Briefly, extracted nucleic acids from 1×10^7^ cells were incubated with the kit assay buffer on cytosine-rich DNA-coated plates for 1 h. 5-Methylcytosine antibody and color development solution were added to the plate, and absorbance was measured using a DTX 880 Multimode Detector (Beckman Coulter Inc., CA, USA); Multimode Analysis Software ver.3.2.0.5 (Beckman Coulter Inc.) was used for the analysis.

### Methylation-sensitive Amplified Fragment Length Polymorphism (MS-AFLP) array

The methylation array was performed as described in a previous report [21]. For preparation of the Not I-Mse I MS-AFLP template, 1 µg of genomic DNA was digested overnight at 37°C with 5 units of the methylation-sensitive restriction enzyme *Not* I (Promega, Madison, WI, USA) and 2 units of methylation-insensitive *Mse* I (NE Biolabs, Beverly, MA, USA). Two pairs of oligonucleotides were annealed overnight at 37°C to generate *Not* I (5′-CTCGTAGACTGCGTAGG-3′ and 5′-GGCCCCTACGCAGTCTAC-3′) and *Mse* I (5′-GACGATGAGTCCTGAG-3′ and 5′-TACTCAGGACTCAT-3′) specific adaptors. The products were then amplified using the Gene Amp PCR System 9700 (Applied Biosystems). The primers used were: Not I: GACTGCGTAGGGGCCGC and Mse I: GATGAGTCCTGAGTAA. Cycle parameters used were: 72°C for 30 sec and 94°C for 30 sec, followed by 36 cycles of 94°C for 45 sec, 52°C for 45 s and 72°C for 2 min, followed by 72°C for 10 min, then reactions were kept at 10 °C. Products were purified using the QIAquick PCR Purification Kit (Qiagen). Fluorescently-labeled probes were prepared using the Bioprime labeling system (Invitrogen). The products were labeled with CY5 Mix solution (dGTP, dATP, dTTP, dCTP and CY5-dCTP) or CY3 Mix solution (dGTP, dATP, dTTP, dCTP and CY3-dCTP) and then hybridized to an array (Agilent Custom Array, Agilent Technologies Japan, Ltd., Tokyo, Japan). Background-corrected signals were used to calculate the log2 ratio of the CY5 vs. CY3 channel for every array feature.

The arrays were scanned using an Agilent G2565BA DNA Microarray Scanner or by Hokkaido System Science Co. Ltd. (Sapporo, Japan), and analyzed using MeV version 4.5. (The arrays used for analysis were the Agilent MouseGenome for mouse tissues, and Agilent HumanGenome for Jurkat cells, Dana-Farber Cancer Institute, MA, USA).

### Statistical analysis

Data were expressed as means ± SD (standard deviation) of several experiments indicated as *n* (number). Comparisons of three groups of data were done using the Kruskal-Wallis test, and comparisons between two groups of data were done with the Mann-Whitney test. A *p* value less than 0.05 was considered to be statistically significant. Survival times were estimated using the Kaplan-Meier method. Comparison of survival between groups was done using the Logrank analysis. Evaluation of tumor formation was tested by Fisher's exact probability test.

## Results and Discussion

**Figure 2:**
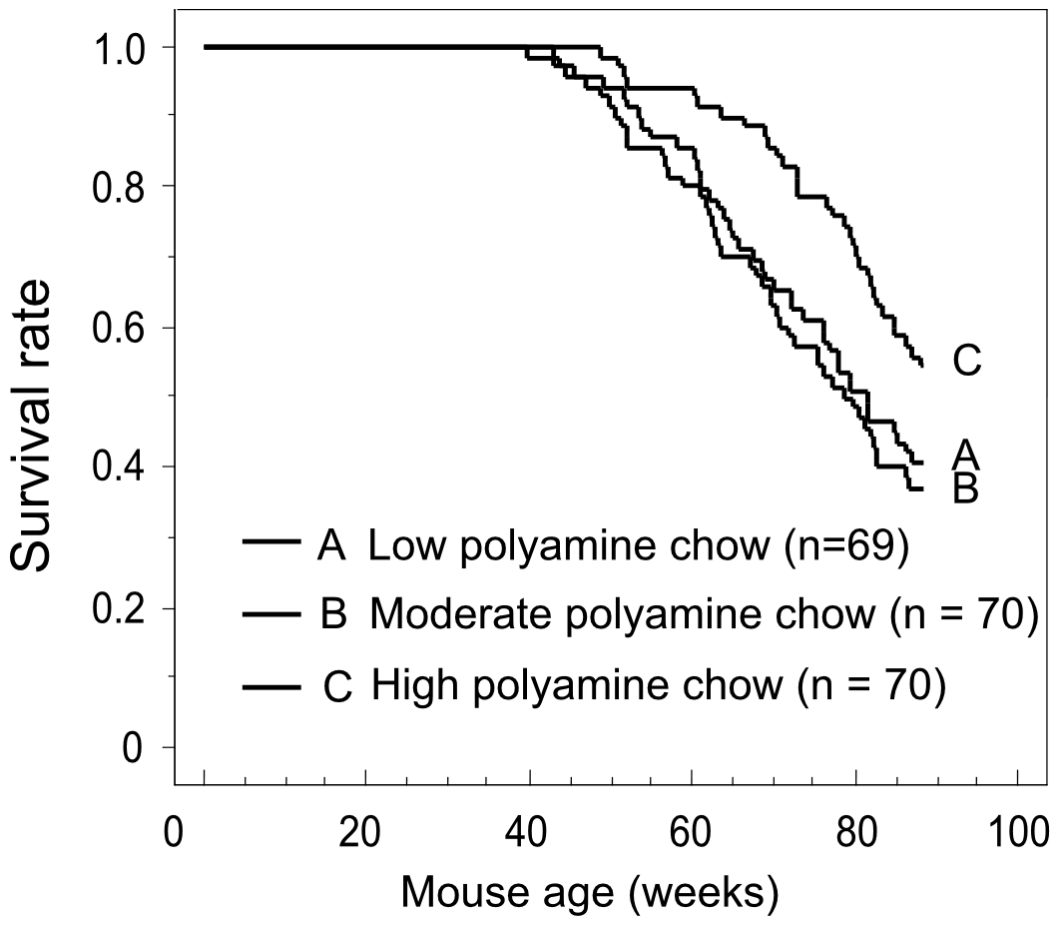
Survival curve. The survival curve was created based on the results of 4 separate experiments. There were significant differences between the moderate and high polyamine chow groups (*p* = 0.010), and between the low and high polyamine groups (*p* = 0.040). No significant difference was found between the low and moderate polyamine groups (*p* = 0.613).

Figure 2 was created using the results of 4 individual experiments combined from the present study and our previous report [9]. Similar to the results of the previous study [9], the lifespan of Jc1:ICR mice fed a high polyamine chow was greater than that of mice fed moderate (Moderate) or low (Low) polyamine chow (Figure 2) (Figure S1). Our previous study showed that the inhibition of age-associated pathological changes was not due to either the decreased food intake or to the associated attenuation of body weight gain, because the body weight was similar among the three groups of mice and the amount of food intake was even greater in mice fed high polyamine chow [9].

**Figure 3:**
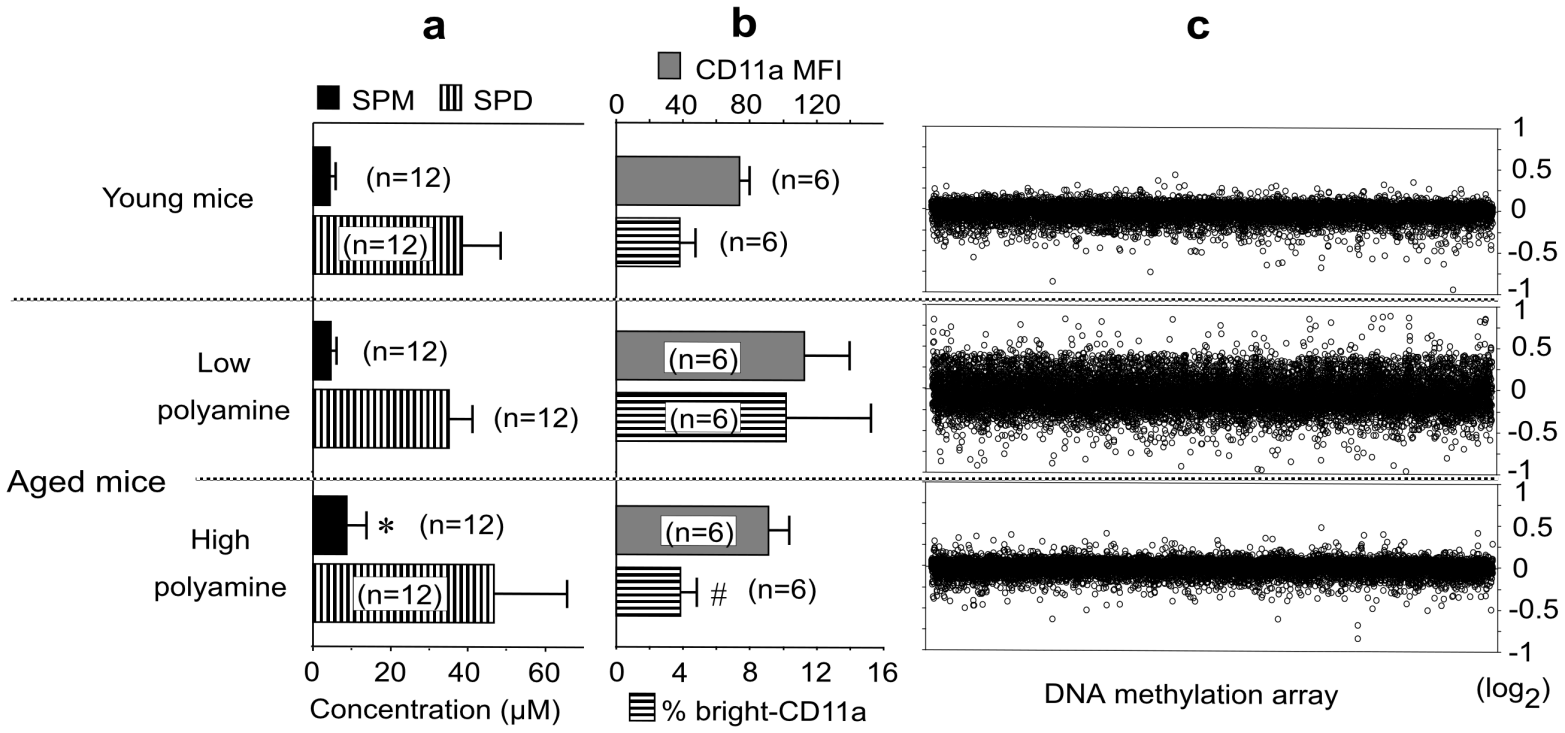
Blood polyamine levels, LFA-1 expression and methylation status. **3-a.** Whole blood polyamine concentration. Polyamine concentrations in whole blood samples from young (24 weeks) and aged mice (80 weeks) were measured by HPLC. * Spermine concentrations in aged mice fed high polyamine chow were significantly higher than in the Low group or in young (24 weeks old) mice (*p* = 0.018 vs. Low, *p* = 0.006 vs. young mice) Bars indicate means and standard deviations. **3-b.** Effect on CD11a expression. ^#^ The percentage of bright CD11a cells in aged mice fed high polyamine chow was significantly (*p* = 0.039) lower than in mice fed low polyamine chow, and was similar to that of young mice. Bars indicate means and standard deviations. **3-c.** Methylation status of kidney genes. MS-AFLP array analysis using a mouse DNA array is shown. Control samples comprised a mixture of tissue from three young mice. Mean data of the log2 ratios of the values from 3 animals relative to the control samples are shown. Positive figures indicate increased demethylation of samples relative to the control, and negative figures indicate increased methylation of samples relative to the control. SPM = Spermine; SPD = Spermidine; Low polyamine = 80 weeks of mice fed low polyamine chow; High = 80 weeks of mice fed high polyamine chow. Young mice = 24 week-old mice.

Whole-blood polyamine levels in the Low group were similar to those in the Moderate group [9], however whole blood spermine concentrations in the High group were increased, with wide inter-individual variation (range 3.40 – 18.80), significantly higher than the Moderate and Low groups, or in young (24 weeks old) mice (*p* = 0.018 vs. Low, *p* = 0.019 vs. Moderate, *p* = 0.006 vs. young mice) (Figure 3a). Although spermidine concentrations in the High group were increased with wide inter-individual variation (range 24.60 – 84.80), they were not significantly different from the Moderate and Low groups, or from young mice (Figure 3a). Similar to our previous study in humans [8], high dietary polyamine intake in mice appears to increase blood levels of spermine to a greater extent than spermidine.

The mean fluorescence intensities (MFIs) of CD11a in lymphocyte and monocyte populations in the young, Low and High groups were 73.90±5.92, 112.62±26.93 and 91.25±12.32, respectively, indicating an age-associated increase in LFA-1 expression (Figure 3b). Although CD11a MFIs in the Low and the High groups were higher than in young mice (*p* = 0.004 vs. the Low, and *p* = 0.025 vs. the High), no significant difference was observed between the Low and High groups (*p* = 0.262). The percentage of bright CD11a cells in the Low group (10.18±5.08%) was significantly higher than in young mice (3.70±1.03%) (*p* = 0.004), and the percentage of bright CD11a cells in the High group (3.84±0.99%) was significantly lower than in the Low group (*p* = 0.039), and was similar to that of young mice (*p* = 0.631). We have previously shown that spermine markedly suppresses LFA-1 expression in human and that spermine potently decreased the number of bright CD11a cells *in vitro* [2]; the present study yielded similar results in the murine model.

At the organ and tissue level, the most significant age-associated pathological changes were observed in the kidney [9]. Preliminary AFLP analyses revealed differences in gene methylation status of several organs between the High and Low groups, and these differences were most significant in kidney. Hence, MS-AFLP was performed using kidney. A kidney methylation microarray in aged mice showed a widespread increase in demethylation and methylation compares to young mice. This age-associated enhancement of genome wide alteration in DNA methylation status seemed to be suppressed by high polyamine intake (Figure 3c) (Dataset S1).

In our previous study, blood spermine concentrations were revealed to have an inverse association with LFA-1 expression in healthy human volunteers, while spermidine had little association with LFA-1 expression [2]. An *in vitro* study showed that although spermidine has biological effects similar to spermine in mammalian cells, the increased intracellular spermidine concentrations were above physiological levels [2]. In addition, previous reports have shown that the importance of polyamines appears to differ among organisms, spermine being more important in mammals than in lower organisms [5], [19], [20], [22]–[24]. Considering these findings and the fact that increased spermine but not spermidine was apparent after increased polyamine intake, we focused on the effects of spermine in our *in vitro* studies.

**Figure 4:**
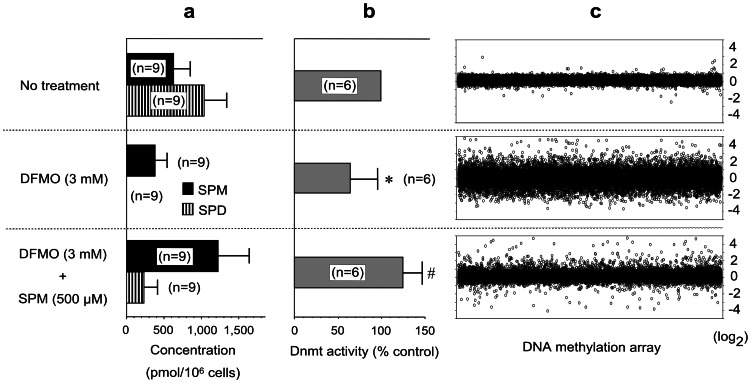
Polyamine concentration, Dnmt activity and methylation status. **4-a.** Polyamine concentration in cells under various conditions. DFMO treatment decreased, and spermine supplementation increased, concentrations of spermine and spermidine in Jurkat cells. Bars indicate means and standard deviations. **4-b.** Effects of polyamine on DNA methyltransferase activity. * DFMO treatment significantly (64.20±28.60% of No treatment cells, *p* = 0.004) decreased, and ^#^ spermine supplementation increased (*p* = 0.004) methyltransferase activities in Jurkat cells. Bars indicate means and standard deviations. **4-c.** Methylation status in Jurkat cells. MS-AFLP array analyses using a human DNA array are shown. DNA from untreated Jurkat cells was used as a control. The log2 ratios of values relative to the control samples are shown. Positive figures indicate increased demethylation of samples relative to the control, and negative figures indicate increased methylation of samples relative to the control. SPM = Spermine; SPD = Spermidine.

As there were significant differences in the histological findings between the High and Low groups, it is possible that the results of the methylation array could be attributable to differences in cell composition. To clarify the effects of polyamines on methylation status, cultured cells were utilized. Inhibition of ODC activity by DFMO decreased spermidine and spermine concentrations (Figure 4a), and DNA methyltransferase (Dnmt) activity in Jurkat cells (64.20±28.60% of control, n = 6, *p* = 0.004) (Figure 4b). DFMO treatment has been shown to increase levels of decarboxylated S-adenosylmethionine (dcSAM) [25], [26], which acts as a competitive inhibitor of methylation reactions (Figure 1). Spermine supplementation increased both spermine and spermidine concentrations (Figure 4a). Since polyamines have play a negative regulatory role in adenosylmethionine decarboxylase (AdoMetDC) synthesis [27], [28], spermine supplementation may decrease dcSAM. To wit, the supplementation in fact activated Dnmt activity (*p* = 0.004, compared to DFMO treatment) (Figure 4b). Increased Dnmt activity induced by 500 µM spermine supplementation (126.90±20.10%, n = 13) was also confirmed using HT-29 cells. In accordance with previous studies in which the inhibition of polyamine synthesis by an ODC inhibitor was found to mediate hypomethylation of CpG islands in genomic DNA and down-regulate Dnmt mRNA and protein expression [29], [30], [31], DFMO treatment resulted in global alteration in DNA methylation status in Jurkat cells. Spermine supplementation inhibited the polyamine-deficient associated global misregulation of DNA methylation status (Figure 4c) (Dataset S2).

ITGAL, a gene that promotes expression of LFA-1, appears to be one target of methylation alteration in response to polyamine metabolism. Decreased Dnmt activity has been reported to be associated with increased demethylation of ITGAL and increased LFA-1 expression [32], [33]. Our recent study showed that increased Dnmt activity induced by polyamine supplementation is accompanied by increased ITGAL methylation and decreased LFA-1 expression [34]. It is known that aging is associated with decreased polyamine synthesis [35], [36], decreased Dnmt activity [37], [38], increased LFA-1 expression [15], [32] and global alteration in DNA methylation status [32], [39]. These findings and the results of the present study suggest that exogenous polyamines play an important role in Dnmt activation, and Dnmt activity seems to maintain the methylation status of the whole genome.

**Figure 5:**
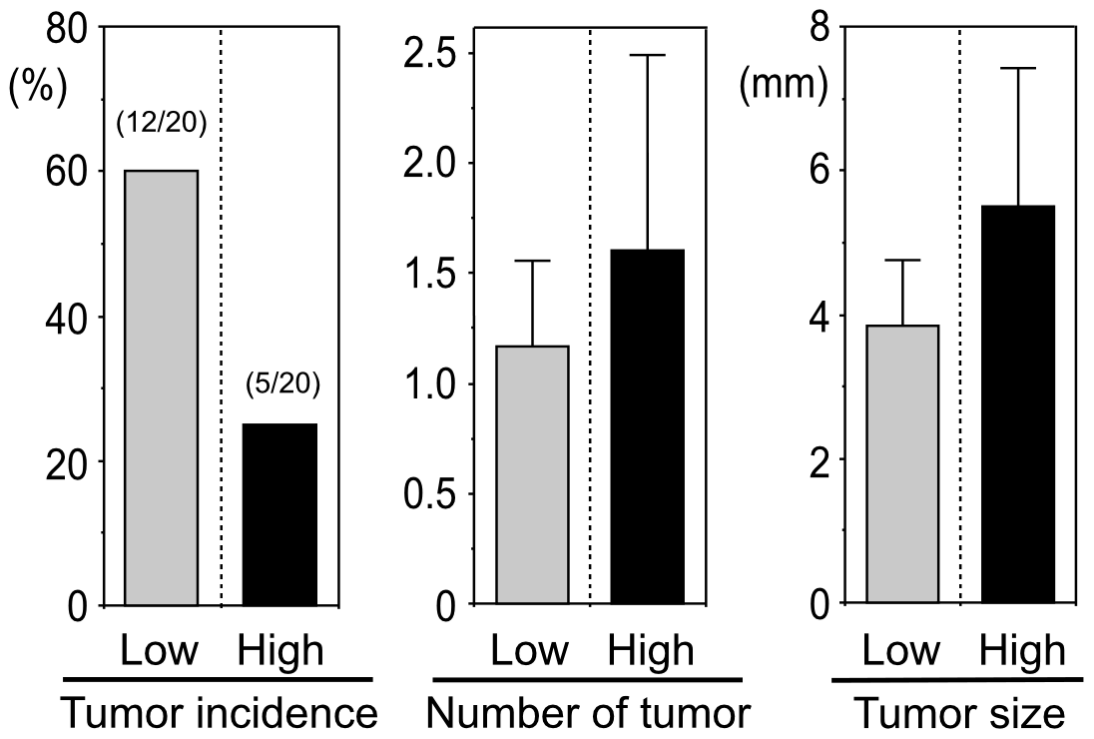
Effect of food polyamine on tumorigenesis. Four-week old male mice (BALB/c) fed either the high polyamine or low polyamine chow were administered 20 mg/kg BW of 1,2-dimethylhydrazine (DMH) (2.0 mg/ml in PBS) subcutaneously for 12 consecutive weeks. Increased polyamine intake suppressed DMH-induced tumorigenesis, but appeared to enhance the growth of established tumors. Low = mice fed low polyamine chow; High = mice fed high polyamine chow.

Global alteration in DNA methylation status associated with age has been documented in many vertebrate tissues [39], [40], and such changes are considered a major cause of neoplastic development and age-associated chronic diseases and fragility [41], [42]. In the present study, in association with inhibition of age-associated pathological changes, inhibition of age-associated alteration in DNA methylation status by increased polyamine intake was accompanied by a decrease in the incidence of 1,2-dimethylhydrazine (DMH)-induced neoplastic development in BALB/c mice. Tumor formation was found in 12 mice (60%) in the Low group, while only 5 mice (25%) had tumors in the High group (Fisher's exact probability = 0.027, *p* = 0.025) (Figure 5). The average number of tumors in mice with detectable tumor formation was 1.17 and 1.60 in the Low and High groups, respectively. Maximal tumor diameter in the Low and High groups was 3.85±0.90 mm and 5.50±1.93 mm, respectively (Mann-Whitney test, *p* = 0.039) (Figure 5). Thus, increased polyamine intake suppresses DMH-induced tumorigenesis, but enhances growth of established tumors. Neoplastic formation was not observed in control PBS-injected mice fed either a Low or High polyamine diet. Increased enzymatic activity required for polyamine synthesis is closely related to neoplastic growth [43]. ODC is a target of oncogenes, and ODC overexpression and the resultant increase in polyamine levels in cells with properties of tumor cells, and in animals with other genetic or epigenetic abnormalities promotes neoplastic transformation [44], [45], [46]. These findings suggest a direct involvement of polyamines in tumorigenesis. However, it has been shown that oncogenic transformation does not occur when wholly normal cells are targeted for ODC overexpression [44], [45], [46]. Increased pro-inflammatory status with age enhances oxidative stress, and increased exposure to oxidative stress leads to epigenetic abnormalities such as global alteration in DNA methylation status leading to neoplastic transformation. Therefore, polyamine-induced suppression of pro-inflammatory status and inhibition of global alteration in DNA methylation status seem to play an important role in inhibiting neoplastic transformation.

## Conclusions

This study, along with finding that healthy foods such as soybeans are rich in polyamines and that healthy dietary patterns such as the Mediterranean diet and Japanese food are associated with increased polyamine content [47], [48], supports the notion of a positive contribution by dietary polyamines to human health and longevity.

## Supporting Information

Dataset S1 The result of methylation microarray of murine kidney.
(XLS)

Dataset S2 The result of methylation microarray of Jurkat cells.
(XLS)

Figure S1 Survival curve in previous and present experiments. Upper) The survival curve was created based on the results of 2 experiments of the previous study. Survival of the High group seemed to be higher than the Low or the Moderate group, however no significant difference was found. Lower) The survival curve was created based on the results of 2 experiments of the present study. While no significant difference was found, the survival of the High group seemed to be higher than other 2 groups of mice.
(TIF)

## References

[1] Zhang M, Caragine T, Wang H, Cohen PS, Botchkina G, et al (1997) Spermine inhibits proinflammatory cytokine synthesis in human mononuclear cells: a counterregulatory mechanism that restrains the immune response. J Exp Med 185: 1759–1768. 9151701 10.1084/jem.185.10.1759 PMC2196317

[2] Soda K, Kano Y, Nakamura T, Kasono K, Kawakami M, et al (2005) Spermine, a natural polyamine, suppresses LFA-1 expression on human lymphocyte. J Immunol 175: 237–245. 15972654 10.4049/jimmunol.175.1.237

[3] Ha HC, Sirisoma NS, Kuppusamy P, Zweier JL, Woster PM, et al (1998) The natural polyamine spermine functions directly as a free radical scavenger. Proc Natl Acad Sci U S A 95: 11140–11145. 9736703 10.1073/pnas.95.19.11140 PMC21609

[4] Fujisawa S, Kadoma Y (2005) Kinetic evaluation of polyamines as radical scavengers. Anticancer Res 25: 965–969. 15868935

[5] Mackintosh CA, Pegg AE (2000) Effect of spermine synthase deficiency on polyamine biosynthesis and content in mice and embryonic fibroblasts, and the sensitivity of fibroblasts to 1,3-bis-(2-chloroethyl)-N-nitrosourea. Biochem J 351 Pt 2: 439–447. PMC1221380 11023830

[6] Newton GL, Aguilera JA, Ward JF, Fahey RC (1996) Polyamine-induced compaction and aggregation of DNA--a major factor in radioprotection of chromatin under physiological conditions. Radiat Res 145: 776–780. 8643839

[7] Beyer HS, Ellefson M, Sherman R, Zieve L (1992) Aging alters ornithine decarboxylase and decreases polyamines in regenerating rat liver but putrescine replacement has no effect. J Lab Clin Med 119: 38–47. 1727906

[8] Soda K, Kano Y, Sakuragi M, Takao K, Lefor A, et al (2009) Long-term oral polyamine intake increases blood polyamine concentrations. J Nutr Sci Vitaminol (Tokyo) 55: 361–366. 19763038 10.3177/jnsv.55.361

[9] Soda K, Dobashi Y, Kano Y, Tsujinaka S, Konishi F (2009) Polyamine-rich food decreases age-associated pathology and mortality in aged mice. Exp Gerontol 44: 727–732. 19735716 10.1016/j.exger.2009.08.013

[10] Brodal BP, Eliassen KA, Ronning H, Osmundsen H (1999) Effects of dietary polyamines and clofibrate on metabolism of polyamines in the rat. J Nutr Biochem 10: 700–708. 15539269 10.1016/s0955-2863(99)00058-3

[11] Wacholtz MC, Patel SS, Lipsky PE (1989) Leukocyte function-associated antigen 1 is an activation molecule for human T cells. J Exp Med 170: 431–448. 2569026 10.1084/jem.170.2.431 PMC2189396

[12] Hibbs ML, Xu H, Stacker SA, Springer TA (1991) Regulation of adhesion of ICAM-1 by the cytoplasmic domain of LFA-1 integrin beta subunit. Science. 251: 1611–1613. 10.1126/science.1672776 1672776

[13] Ding ZM, Babensee JE, Simon SI, Lu H, Perrard JL, et al (1999) Relative contribution of LFA-1 and Mac-1 to neutrophil adhesion and migration. J Immunol 163: 5029–5038. 10528208

[14] Werther WA, Gonzalez TN, O'Connor SJ, McCabe S, Chan B, et al (1996) Humanization of an anti-lymphocyte function-associated antigen (LFA)-1 monoclonal antibody and reengineering of the humanized antibody for binding to rhesus LFA-1. J Immunol 157: 4986–4995. 8943405

[15] Chiricolo M, Morini MC, Mancini R, Beltrandi E, Belletti D, et al (1995) Cell adhesion molecules CD11a and CD18 in blood monocytes in old age and the consequences for immunological dysfunction. Preliminary results. Gerontology 41: 227–234. 7557500 10.1159/000213686

[16] Powers DC, Morley JE, Flood JF (1992) Age-related changes in LFA-1 expression, cell adhesion, and PHA-induced proliferation by lymphocytes from senescence-accelerated mouse (SAM)-P/8 and SAM-R/1 substrains. Cell Immunol 141: 444–456. 1576658 10.1016/0008-8749(92)90162-i

[17] Franceschi C, Bonafe M, Valensin S, Olivieri F, De Luca M, et al (2000) Inflamm-aging. An evolutionary perspective on immunosenescence. Ann N Y Acad Sci 908: 244–254. 10911963 10.1111/j.1749-6632.2000.tb06651.x

[18] Eisenberg T, Knauer H, Schauer A, Buttner S, Ruckenstuhl C, et al (2009) Induction of autophagy by spermidine promotes longevity. Nat Cell Biol 11: 1305–1314. 19801973 10.1038/ncb1975

[19] Lyon MF, Scriver CR, Baker LR, Tenenhouse HS, Kronick J, et al (1986) The Gy mutation: another cause of X-linked hypophosphatemia in mouse. Proc Natl Acad Sci U S A 83: 4899–4903. 3460077 10.1073/pnas.83.13.4899 PMC323851

[20] Wang X, Ikeguchi Y, McCloskey DE, Nelson P, Pegg AE (2004) Spermine synthesis is required for normal viability, growth, and fertility in the mouse. J Biol Chem 279: 51370–51375. 15459188 10.1074/jbc.M410471200

[21] Yamamoto F, Yamamoto M (2004) A DNA microarray-based methylation-sensitive (MS)-AFLP hybridization method for genetic and epigenetic analyses. Mol Genet Genomics 271: 678–686. 15146327 10.1007/s00438-004-1017-5

[22] Chattopadhyay MK, Tabor CW, Tabor H (2003) Spermidine but not spermine is essential for hypusine biosynthesis and growth in Saccharomyces cerevisiae: spermine is converted to spermidine in vivo by the FMS1-amine oxidase. Proc Natl Acad Sci U S A 100: 13869–13874. 14617780 10.1073/pnas.1835918100 PMC283513

[23] Hamasaki-Katagiri N, Katagiri Y, Tabor CW, Tabor H (1998) Spermine is not essential for growth of Saccharomyces cerevisiae: identification of the SPE4 gene (spermine synthase) and characterization of a spe4 deletion mutant. Gene 210: 195–201. 9573363 10.1016/s0378-1119(98)00027-4

[24] Lorenz B, Francis F, Gempel K, Boddrich A, Josten M, et al (1998) Spermine deficiency in Gy mice caused by deletion of the spermine synthase gene. Hum Mol Genet 7: 541–547. 9467015 10.1093/hmg/7.3.541

[25] Mamont PS, Danzin C, Wagner J, Siat M, Joder-Ohlenbusch AM, et al (1982) Accumulation of decarboxylated S-adenosyl-L-methionine in mammalian cells as a consequence of the inhibition of putrescine biosynthesis. Eur J Biochem 123: 499–504. 6804235 10.1111/j.1432-1033.1982.tb06559.x

[26] Danzin C, Claverie N, Wagner J, Grove J, Koch-Weser J (1982) Effect on prostatic growth of 2-difluoromethylornithine, an effective inhibitor of ornithine decarboxylase. Biochem J 202: 175–181. 6177318 10.1042/bj2020175 PMC1158088

[27] Mamont PS, Joder-Ohlenbusch AM, Nussli M, Grove J (1981) Indirect evidence for a strict negative control of S-adenosyl-L-methionine decarboxylase by spermidine in rat hepatoma cells. Biochem J 196: 411–422. 6797404 10.1042/bj1960411 PMC1163012

[28] Porter CW, Pegg AE, Ganis B, Madhabala R, Bergeron RJ (1990) Combined regulation of ornithine and S-adenosylmethionine decarboxylases by spermine and the spermine analogue N1 N12-bis(ethyl)spermine. Biochem J 268: 207–212. 2344358 10.1042/bj2680207 PMC1131413

[29] Yamamoto D, Shima K, Matsuo K, Nishioka T, Chen CY, et al (2010) Ornithine decarboxylase antizyme induces hypomethylation of genome DNA and histone H3 lysine 9 dimethylation (H3K9me2) in human oral cancer cell line. PLoS One 5: e12554. 20838441 10.1371/journal.pone.0012554 PMC2933235

[30] Papazafiri P, Osborne HB (1987) Effect of alpha-difluoromethylornithine on DNA methylation in murine erythroleukaemic cells. Relationship to stimulation of induced differentiation. Biochem J 242: 479–483. 3109392 10.1042/bj2420479 PMC1147730

[31] Frostesjo L, Holm I, Grahn B, Page AW, Bestor TH, et al (1997) Interference with DNA methyltransferase activity and genome methylation during F9 teratocarcinoma stem cell differentiation induced by polyamine depletion. J Biol Chem 272: 4359–4366. 9020157 10.1074/jbc.272.7.4359

[32] Zhang Z, Deng C, Lu Q, Richardson B (2002) Age-dependent DNA methylation changes in the ITGAL (CD11a) promoter. Mech Ageing Dev 123: 1257–1268. 12020947 10.1016/s0047-6374(02)00014-3

[33] Lu Q, Ray D, Gutsch D, Richardson B (2002) Effect of DNA methylation and chromatin structure on ITGAL expression. Blood 99: 4503–4508. 12036881 10.1182/blood.v99.12.4503

[34] Kano Y. Soda K, Konishi F (2013) Suppression of LFA-1 Expression by Spermine Is Associated with Enhanced Methylation of ITGAL, the LFA-1 Promoter Area. PLoS One 8: e56056. Available: http://www.plosone.org/article/info%3Adoi%2F10.1371%2Fjournal.pone.0056056 Accessed 2013 Feb 13. 23418509 10.1371/journal.pone.0056056 PMC3572138

[35] Shain SA, Schultz JJ, Lancaster CM (1986) Aging in the AXC/SSh rat: diminished prostate L-ornithine decarboxylase (ODC) activity reflects diminished prostate ODC protein and transcript content. Endocrinology 119: 1830–1838. 2428603 10.1210/endo-119-4-1830

[36] Das R, Kanungo MS (1982) Activity and modulation of ornithine decarboxylase and concentrations of polyamines in various tissues of rats as a function of age. Exp Gerontol 17: 95–103. 7106211 10.1016/0531-5565(82)90042-0

[37] Lopatina N, Haskell JF, Andrews LG, Poole JC, Saldanha S, et al (2002) Differential maintenance and de novo methylating activity by three DNA methyltransferases in aging and immortalized fibroblasts. J Cell Biochem 84: 324–334. 11787061 10.1002/jcb.10015

[38] Romanenko EB, Demidenko ZN, Vanyushin BF (1998) RNA-polymerase, DNA-polymerase, DNA-methyltransferase and sphingomyelinase activities in liver nuclei of rats of different Age. Biochemistry (Mosc) 63: 159–163. 9526108

[39] Vanyushin BF, Tkacheva SG, Belozersky AN (1970) Rare bases in animal DNA. Nature 225: 948–949. 4391887 10.1038/225948a0

[40] Wilson VL, Smith RA, Ma S, Cutler RG (1987) Genomic 5-methyldeoxycytidine decreases with age. J Biol Chem 262: 9948–9951. 3611071

[41] White R, Parker M (1983) Developmental changes in DNA methylation around prostatic steroid-binding protein genes. J Biol Chem 258: 8943–8948. 6306005

[42] Ono T, Uehara Y, Kurishita A, Tawa R, Sakurai H (1993) Biological significance of DNA methylation in the ageing process. Age Ageing 22: S34–43. 8438654 10.1093/ageing/22.suppl_1.s34

[43] Russell DH (1983) Clinical relevance of polyamines. Crit Rev Clin Lab Sci 18: 261–311. 6339165 10.3109/10408368209085073

[44] Clifford A, Morgan D, Yuspa SH, Soler AP, Gilmour S (1995) Role of ornithine decarboxylase in epidermal tumorigenesis. Cancer Res 55: 1680–1686. 7712475

[45] Hibshoosh H, Johnson M, Weinstein IB (1991) Effects of overexpression of ornithine decarboxylase (ODC) on growth control and oncogene-induced cell transformation. Oncogene 6: 739–743. 1711188

[46] O'Brien TG, Megosh LC, Gilliard G, Soler AP (1997) Ornithine decarboxylase overexpression is a sufficient condition for tumor promotion in mouse skin. Cancer Res 57: 2630–2637. 9205069

[47] Binh PNT, Soda K, Kawakami M (2010) Mediterranean diet and polyamine intake –possible contribution of increased polyamine intake to inhibition of age-associated disease. Nutrition and Dietary Supplements 3: 1–7.

[48] Binh PNT, Soda K, Maruyama C, Kawakami M (2010) Relationship between food polyamines and gross domestic product in association with longevity in Asian countries. Health 2: 1390–1396.

