# Supplementary figures and images for: Increased Polyamine Intake Inhibits Age-Associated Alteration in Global DNA Methylation and 1,2-Dimethylhydrazine-Induced Tumorigenesis
Source: PLoS One. 2013 May 16;8(5):e64357. doi: 10.1371/journal.pone.0064357 (PMC3655973; doi:10.1371/journal.pone.0064357)

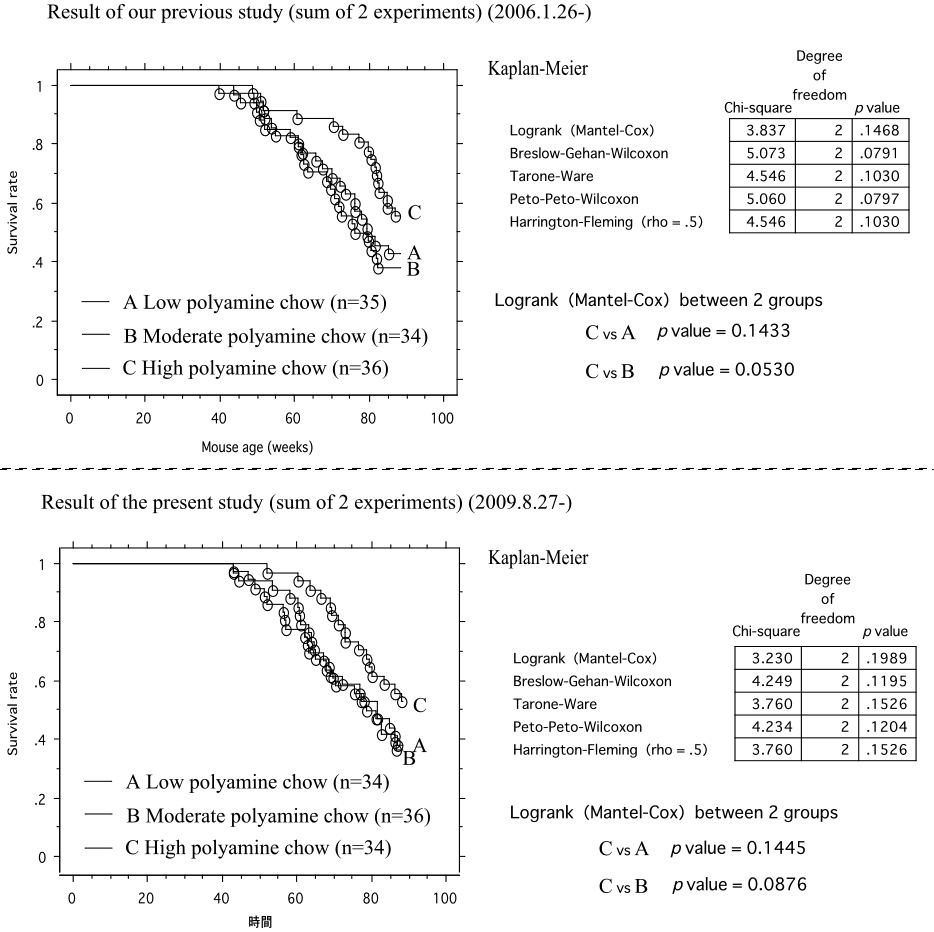

Supplement: Figure S1 — Survival curve in previous and present experiments. Upper) The survival curve was created based on the results of 2 experiments of the previous study. Survival of the High group seemed to be higher than the Low or the Moderate group, however no significant difference was found. Lower) The survival curve was created based on the results of 2 experiments of the present study. While no significant difference was found, the survival of the High group seemed to be higher than other 2 groups of mice. (TIF) [file pone.0064357.s003.tif]
